# Vancomycin pharmacokinetic model development in patients on intermittent online hemodiafiltration

**DOI:** 10.1371/journal.pone.0216801

**Published:** 2019-05-14

**Authors:** Niels Westra, Johannes H. Proost, Casper F. M. Franssen, Erik B. Wilms, Marjolijn van Buren, Daan J. Touw

**Affiliations:** 1 University of Groningen, University Medical Center Groningen, Department of Clinical Pharmacy and Pharmacology, Groningen, The Netherlands; 2 University of Groningen, University Medical Center Groningen, Groningen, The Netherlands; 3 University of Groningen, University Medical Center Groningen, department of Nephrology, Groningen, The Netherlands; 4 Pharmacy Haagse Ziekenhuizen, Den Haag, The Netherlands; 5 HagaZiekenhuis, Den Haag, The Netherlands; Universidade Estadual Paulista Julio de Mesquita Filho, BRAZIL

## Abstract

**Background:**

Vancomycin is frequently used in hemodialysis (HD) and in hemodiafiltration (HDF) patients and is usually administered in the last 30 or 60 minutes of a dialysis session. Vancomycin pharmacokinetics are not well described in HDF patients. The aim of this study is to develop a population pharmacokinetic (PPK) model and dosing regimen for vancomycin in HDF patients and to evaluate its applicability in low-flux (LF-HD) patients.

**Methods:**

Two-compartment PPK models were developed using data from HDF patients (n = 17), and was parameterized as follows: non-renal clearance (CLm), renal clearance as a fraction of creatinine clearance (fr), central volume of distribution (V1), intercompartmental clearance (CL12), peripheral volume of distribution (V2) and extracorporeal extraction ratio (Eec). We evaluated the final model in a cohort of LF-HD patients (n = 21). Dosing schemes were developed for a vancomycin 24-h AUC of 400 mg*h/L.

**Results:**

Model parameters (± SD) were: CLm = 0.473 (0.271) L/h, fr = 0.1 (fixed value), V1 = 0.278 (0.092) L/kgLBMc, CL12 = 9.96 L/h (fixed value), V2 = 0.686 (0.335) L/kgLBMc and Eec = 0.212 (0.069). The model reliably predicted serum levels of vancomycin in both HDF and LF-HD patients during and between dialysis sessions. The median of the prediction error (MDPE) as a measure of bias is -0.7% (95% CI: -3.4%-1.7%) and the median of the absolute values of the prediction errors (MDAPE) as a measure of precision is 7.9% (95% CI: 6.0%-9.8%). In both HDF and LF-HD, the optimal vancomycin loading dose for a typical patient weighing 70 kg is 1700 mg when administered during the last 60 minutes of the hemodialysis session. Maintenance dose is 700 mg if administered during the last 30 or 60 minutes of the hemodialysis session.

**Conclusion:**

The developed PPK model for HDF is also capable of predicting serum levels of vancomycin in patients on LF-HD. A dosing regimen was developed for the use of vancomycin in HDF and LF-HD.

## Introduction

Dialysis patients are at an increased risk for diseases and complications due to infections[1]. The annual death rate in dialysis patients due to sepsis is 100–300 times higher than in the general population[1].Vancomycin is frequently used in patients on intermittent hemodialysis to treat infections with gram-positive micro-organisms like Staphylococcus epidermidis and Staphylococcus aureus[2]. For patients convenience, most centers administer vancomycin in the last 30 or 60 minutes of the hemodialysis session and not on interdialysis days.

The efficacy of vancomycin is associated with the area under the serum concentration—time curve (AUC)[3]. Reviews suggests that the AUC divided by the Minimum Inhibitory Concentration (MIC) best correlates with a successful outcome[4]. Adequate AUC/MIC ratios are important to prevent selection of resistant organisms and to improve the efficacy[5]. Therapeutic Drug Monitoring (TDM) aiming at a target AUC_24h_/MIC ≥400 mg*h/L is generally used for designing and optimizing dosing regimens in patients treated with vancomycin[3]. In clinical practice vancomycin is used up till a MIC of 1mg/L so clinicians aim at an AUC_24h_ ≥400 mg*h/L. For optimal guidance, population pharmacokinetic (PPK) models are used to calculate the optimal initial dose with subsequently optimization of vancomycin exposure using Bayesian therapeutic drug monitoring (TDM)[6].

Vancomycin is predominantly cleared by the kidneys[4]. In dialysis patients renal clearance of vancomycin is strongly reduced. Vancomycin is removed by hemodialysis, but vancomycin can be administered during the last 30 or 60 minutes of the hemodialysis session if the vancomycin dose is augmented with the amount cleared by dialysis. The vancomycin clearance of the dialyzer is substantial and was reported to vary between 9.6 and 130.7 ml/min for low- and high-flux hemodialysis patients[7]. A dialysis patient is also prone to altered pharmacokinetic parameters like distribution, metabolism and other elimination processes which underlines the use of serum concentrations to enable adequate therapy[3].

Online hemodiafiltration (HDF) is increasingly used in the outpatient setting as method of hemodialysis[3]. HDF is basically a combination of hemodialysis and hemofiltration using the (physical) principles of both diffusion and convection[8]. Low molecular weight molecules are effectively cleared by diffusion[3]. Convection however is less dependent on molecular weight, so due to the convection component of HDF the clearance of large molecules is improved in HDF compared to HD[3]. Jager et al. concluded that larger molecules (defined as >500 Da), like vancomycin (1450 Da[4]), are likely to be cleared more effectively by HDF compared to high-flux hemodialysis (HF-HD)[3]. Ghouti-Terki et al. suggested that vancomycin clearance in HDF patients is probably increased compared with HF-HD[9]. However, their opinion is based on a study in only 2 HDF patients, so any firm conclusion about the potentially increased clearance cannot be drawn[9].

Pharmacokinetic data for dosing vancomycin during HDF is lacking. Because of this paucity, the aim of our study was 1) to develop a PPK model for vancomycin in HDF patients and 2) to evaluate the predictive performance of this model in low-flux hemodialysis (LF-HD) patients to investigate if there indeed is a difference in vancomycin pharmacokinetics in modern HDF compared to modern LF-HD and 3) to develop an ‘a priori’ dosing scheme for vancomycin in HDF and in LF-HD patients that can subsequently be used for TDM with Bayesian feedback to further optimize the vancomycin exposure.

## Materials and methods

### Patient data

This observational and retrospective study was performed at the HagaZiekenhuis, The Hague, The Netherlands and the University Medical Center Groningen (UMCG), Groningen, The Netherlands. The data from HagaZiekenhuis (n = 17 online HDF patients) were used to develop the model, the data from UMCG (n = 21 low-flux HD patients) were subsequently used to evaluate the model.

Patients receiving vancomycin by intravenous infusion and with more than 1 extracorporeal clearance period (performed as online HDF) between January 1^st^, 2002 and January 1^st^, 2007 were included at the HagaZiekenhuis. Patients receiving vancomycin by intravenous infusion and with more than 1 extracorporeal clearance period (performed as LF-HD) between January 1^st^, 2009 and December 31^th^, 2016 were included at the UMCG. In both hospitals, vancomycin was given during the last 30 or 60 minutes of the dialysis session. In both cohorts the following patient characteristics were obtained from the (electronic) patient charts: age, weight, height, gender, vancomycin time of administration, vancomycin infusion time, vancomycin dose, route of administration, vancomycin serum levels, dialysis type (LF-HD or HDF), dialysis start/stop times, dialyzer filter type, plasma creatinine levels, 24 h urine excretion of creatinine, plasma urea levels and 24h urine excretion of urea. Patients with missing date of any of these parameters were excluded. If the same patient had multiple vancomycin courses, only the first course of therapy was included in this study.

Residual renal function was estimated using the plasma creatinine and urea levels, and 24 h urine excretion of creatinine and urea (according to the U*V/P formula shown in Eq 1)[10].

$$Renal\;function\left(\frac{ml}{\text{min}}\right)=\;0.5\times \left(\left(\frac{24h\;creatinine\;urine\left(mmol\right)\times \frac{1000}{24\;h\;\times 60\;min}}{Serum\;creatinine\left(\frac{mmol}{l}\right)}\right)+\left(\frac{24h\;urea\;urine\left(mmol\right)\times \frac{1000}{24\;h\;\times 60\;min}}{Serum\;urea\left(\frac{mmol}{l}\right)}\right)\right)$$ (1)

Vancomycin levels were drawn at the start of dialysis, during dialysis but shortly before vancomycin administration and immediately after the end of dialysis after vancomycin administration. The patients in the development cohort (HDF patients) were dialyzed using FX80 high-flux filters [Fresenius Medical Care Nederland B.V., Nieuwkuijk, the Netherlands]. The patients in the evaluation cohort (LF-HD) were dialyzed using either Polyflux 14L [Baxter Nederland B.V., Utrecht, the Netherlands], Polyflux 17L [Baxter Nederland B.V., Utrecht, the Netherlands], Polyflux 21L [Baxter Nederland B.V., Utrecht, the Netherlands] or Sureflux 15 UX [Nipro Europe N.V., Zaventem, Belgium].

### Ethical considerations

The board of directors of the HagaZiekenhuis has approved the use of the anonymised data. Because of the retrospective nature of this study, a waiver for the use of the UMCG data was obtained for this study from the medical ethical committee in the UMCG according to the act about Medical Research Involving Human Subjects (in Dutch: WMO) [date: December 13, 2016; file reference: M16.204398].

### Vancomycin assay

All vancomycin levels in serum were determined using the same immunoassay technology (PETINA, performed on an Abbot Architect C8000 platform). The assay error, was described by the following equation: SD = 1.3842+0.0626×C+0.0018×C^2^, where C is the vancomycin concentration in mg/L.

### Model development

All modeling and model evaluations were carried out using the MW\Pharm 3.83 pharmacokinetic modeling software (Mediware, Groningen, the Netherlands)[11]. The KinPop module in MW\Pharm 3.83 was used for iterative two stage Bayesian (ITSB) modeling[11].

Two-compartment models were constructed using different parameter settings: iterative Bayesian analysis (“Bayesian”), a predefined fixed population value and standard deviation (fixed population Bayesian, “FPB”), or set to a fixed value (“Fixed”). The developed models were compared with each other using the Akaike information criterion (AIC) and the weighted sum of squares of the residuals (∑WSS) divided by the degrees of freedom (df), where the best model is selected based on the lowest values of AIC and ∑WSS/df.

The first step was to develop a naive base PPK model without covariates and stepwise adding different covariates, the covariate with the lowest AIC was used in the next steps. The next step in developing the model was to set all parameters fixed to literature values[9] and one parameter at a time was changed to either Bayesian or FPB. The parameter in setting Bayesian or FPB with the lowest AIC was chosen for the next step, a drop in AIC of 2 or more was considered as a threshold for a better model[12]. The new parameter and setting from the previous step were used for the next parameterization step and again the setting of one parameter at a time was changed. Again, the setting with the lowest AIC was used in the next step. If no improvement of AIC was obtained with either “Bayesian” or “FPB”, the parameter value remained “Fixed”. This was continued until no significant improvement of AIC was observed, compared to the previous step. The final settings were checked again using AIC and ∑WSS/df to see if the optimal settings for all the parameters were chosen.

Since vancomycin behaves as a 2-compartment model, 2-compartment models were developed with estimates for non-renal clearance (CLm), renal vancomycin clearance as a fraction of creatinine clearance (fr), central volume of distribution (V1), intercompartmental clearance (CL12), peripheral volume (V2) and extracorporeal extraction ratio (Eec). The total clearance in the model was calculated according to Eq 2, were Qec is the extracorporeal bloodflow and Eec is the extracorporeal extraction ratio.

CL=CLm+fr*CLcr+Qec*Eec (2)

With the exception of dialysis sessions the Qec is zero and does not influence the clearance outside the hemodialysis sessions. Our PPK model had the lowest AIC if V1 and V2 were corrected for fat distribution using lean body mass corrected (kgLBMc) according to Eq 3[13]. We only tested LMBc as a covariate because this is the default covariate in MW\Pharm and we deemed our population too small for doing a full covariate analysis.

LBMc=LBM+ffat*(BW−LBM) (3)

Where LBM is lean body mass, BW is body weight (kg) and *ffat* distribution over fat factor and was set to 0.4[14]. LBM was calculated by 50.0 + 0.9 * (Height (cm)– 152) for male patients and 45.5 + 0.9 * (height(cm)– 152) for female patients[15].

Inter-individual variability of the pharmacokinetic parameters was assumed to be log-normally distributed.

The η-shrinkage of the parameters of the final model was calculated according to 1-SD_ind_/SD_pop_[16]. SD_ind_ is the standard deviation of the individual values and SD_pop_ is the standard deviation of the population values.

A goodness-of-fit plot was constructed by plotting the individual and population predicted vancomycin serum levels using the final model against the actually measured vancomycin serum levels. The individual predicted concentrations were calculated using the KinPop module in MW\Pharm set to one cycle. In this setting MW\Pharm calculates the individual parameters without changing the population parameters. The population predicted concentrations were calculated by fixing all parameters to the final model parameters, in this manner MW\Pharm calculates the population predicted concentrations. To evaluate the robustness of the final model, a bootstrap analysis was performed. 1000 replicate sets of the population were generated[12]. The replicate parameter estimates were tabulated and the lower 2.5% and upper 97.5% value of each parameter were estimated to obtain the nonparametric 95% confidence interval (CI).

### Evaluating the model in a LF-HD population

The predictive performance of the developed model was evaluated in a cohort of LF-HD patients (n = 21). The individual predicted concentrations were calculated using the KinPop module in MW\Pharm set to one cycle using the final PPK model. The population predicted concentrations were calculated by fixing all parameters to the final model parameters.

A goodness-of-fit plot was constructed by plotting the individual and population predicted values using the developed model against the measured concentrations. The median of the prediction error (MDPE) was calculated as a measure of bias and the median of the absolute values of the prediction errors (MDAPE) was calculated as a measure of precision. The MDPE for individual predicted concentrations was calculated according to Eq 4 and the MDAPE for individual predicted concentrations was calculated according to Eq 5. The upper and lower nonparametric CI of the MDPE and MDAPE were obtained by bootstrap analysis with 10000 repetitions.

$$MDPE\;=\;median\left(\frac{Cpredicted-Cobserved}{Cobserved}\right)$$ (4)

$$MDAPE=\;median\left|\frac{Cpredicted-Cobserved}{Cobserved}\right|$$ (5)

Furthermore the weighted residuals were calculated and plotted in a residuals plot.

### Dosing regimen

**Fig 1:**
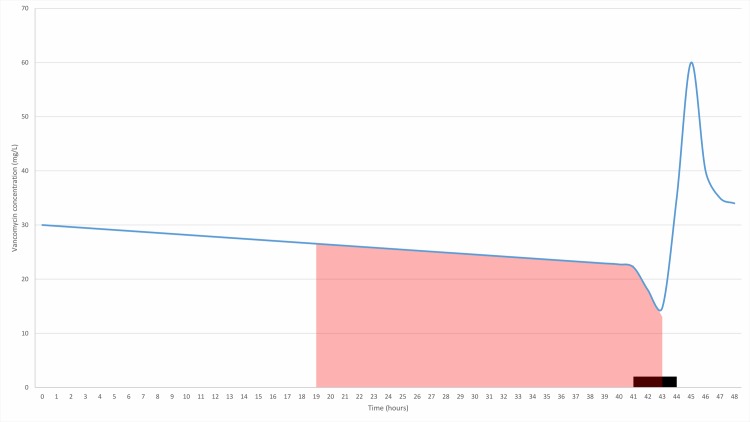
Visual representation of the targeted AUC24h. The shaded area represents the AUC_24h_ (mg*h/L) in the worst-case scenario, that is the 24 hours preceding the administration of vancomycin during dialysis session. The black area represent the dialysis session of 4 hours. Vancomycin was administered in the final 60 minutes of the dialysis session.

To develop a dosing regimen for the ‘a priori’ dose of vancomycin for any patient on HDF, different ‘standard patients’ with different characteristics were simulated with MW\Pharm. For all simulated patients a residual CLcr of 4.4 ml/min was assumed. Because the MIC levels in both populations were not known, we assumed a MIC of 1 mg/L and therefore the AUC_24h_ had to be above and as close as possible to 400 mg*h/L and vancomycin could only be dosed during hemodialysis sessions. The AUC_24h_≥400 mg*h/L was simulated in the worst-case scenario, that is the 24 hours preceding the administration of vancomycin during the dialysis session, including the steep drop in vancomycin serum levels due to dialysis. Fig 1 shows a visual representation of this worst-case scenario. ‘Standard patients’ were created receiving vancomycin during the last 30 versus the 60 minutes with Qec of 200, 250 and 300 ml/min with different weight classes of 50, 60, 70, 80, 90, 100, 110 and 120 kg. Since dialysis usually is carried out 3 times weekly, dosing intervals of 48 h and 72 h were applied. Because this dosing regimen is designed to be used in clinical settings, dosage of vancomycin was rounded up to the nearest multiple of 100 mg vancomycin.

### Statistical analysis

Continuous variables were tested with Levene’s test for equality of variances and a t-test for equality of means, with a limit of significance of P <0.05. All tests were carried out using IBM SPSS Statistics for Windows 23.0 (IBM Corp, Armonk, NY, USA).

## Results

### Patient data

Initially the development cohort consisted of 152 unique patients and the evaluation cohort of 58 unique patients. In the development cohort 135 patients were excluded and in the evaluation cohort 37 patients were excluded because of missing data (e.g. bodyweight, height, creatinine levels and urea levels). The demographic data after patient selection and the clinical characteristics of the development cohort (n = 17, with 159 vancomycin samples) and the evaluation cohort (n = 21, with 132 vancomycin samples) are shown in Table 1.

**Table 1:** Demographic data and clinical characteristics of the development and evaluation cohorts.

Characteristics	Development cohort (n = 17, 159 vancomycin samples)			Evaluation cohort (n = 21, 132 vancomycin samples)			
	Median	Mean	Range	Median	Mean	Range	P-value
**Age (years)**	63	64.8	46–85	66	64.4	39–78	0.93
**Weight (kg)**	72.9	74.9	53–95.7	70	74.0	38–133	0.88
**Height (cm)**	165	165.2	145–189	173	170.0	145–190	0.20
**Gender**	9M/8F	9M/8F		15M/6F	15M/6F		
**CLcr (ml/min)**	2.9	4.4	0.4–16.5	2.8	3.3	0.07–9.6	0.40
**Number of vancomycin samples per patient**	8	9.4	2–24	4	6.3	1–20	0.15

### Model development

**Fig 2:**
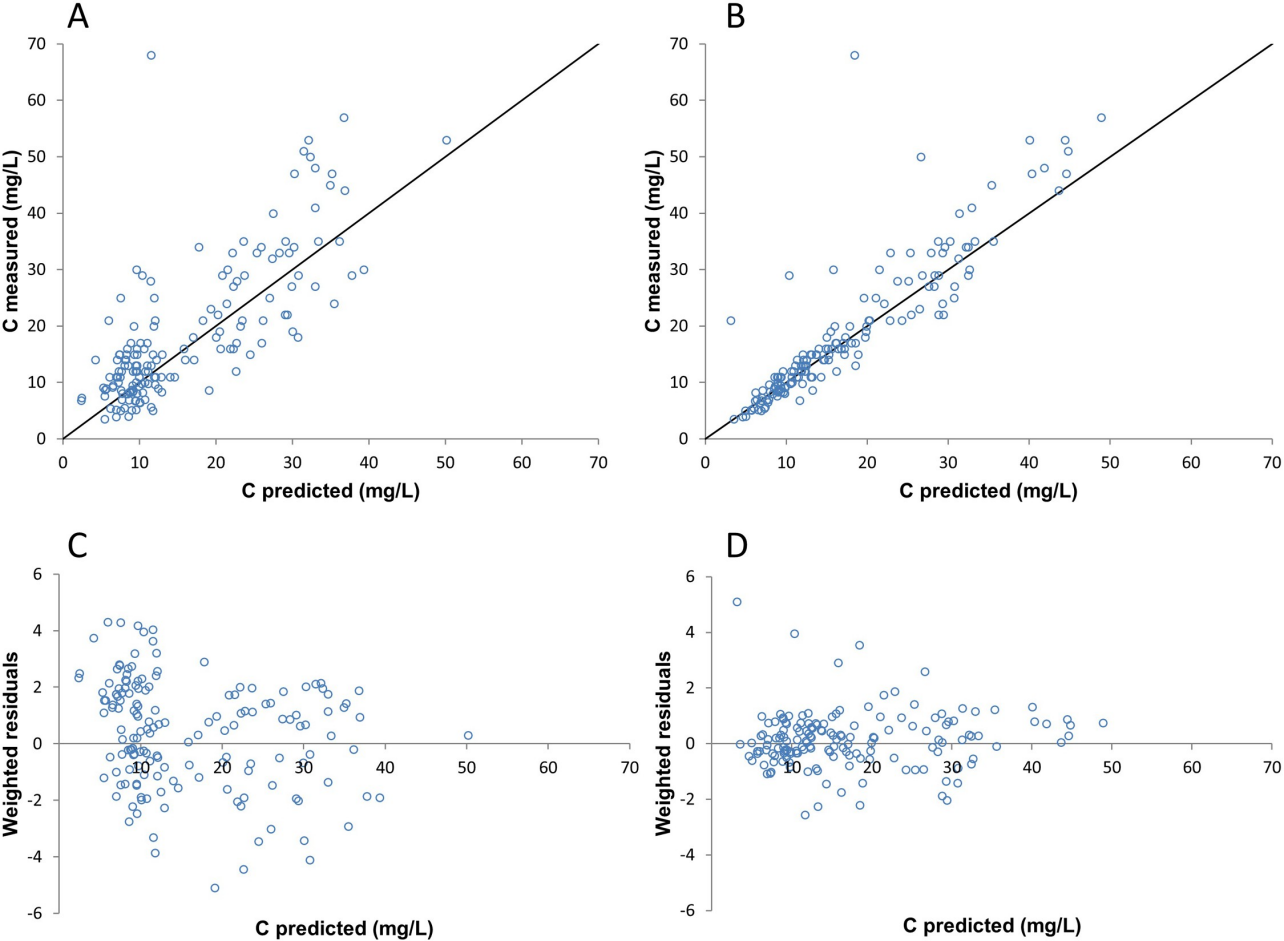
Diagnostic goodness of fit and weighted residuals plots of the development cohort (HDF patients). (A) Population predicted vancomycin serum levels based on the final model versus the actually measured vancomycin serum levels. (B) Individual predicted vancomycin serum levels based on the final model versus the actually measured vancomycin serum levels. (C) Weighted residuals of the population predicted vancomycin serum levels versus the predicted vancomycin serum levels. (D) Weighted residuals of the individual predicted vancomycin serum levels versus the predicted vancomycin serum levels.

In the final model fr and CL12 were fixed on respectively 0.1 and 9.96 L/h, CLm, V1, V2 and Eec were estimated using iterative Bayesian analysis. Table 2 shows the final population parameters for vancomycin during HDF, for CLm, V1, V2 and Eec the η-shrinkage and 95% confidence interval (obtained by bootstrap with 1000 repetitions) is also shown in Table 2. Fig 2A and 2B shows the goodness-of-fit plot for the population and individual predicted vancomycin concentrations respectively. The data point in the individual predicted vancomycin concentrations in Fig 2B are closer distributed along the line of identity compared to the population predicted concentrations of vancomycin in Fig 2A (HDF patients). The weighted residuals of the individual predicted concentrations of vancomycin in Fig 2D are also closer distributed along the line of identity, compared to the weighted residuals of the population predicted concentrations of vancomycin in Fig 2C.

**Table 2:** Population parameters for vancomycin during online HDF dialysis. (the nonparametric 95% CI is obtained by bootstrapping with 1000 repetitions).

PK parameter		Final model	η-shrinkage (%)	CI 95%
**CLm(L/h)**	Mean	0.473		[0.291; 0.633]
	SD	0.271	9.1	[0.090; 0.328]
**Fr(-)**	Mean	0.100 (fixed)		
	SD	0		
**V1(L/kgLBMc)**	Mean	0.278		[0.232; 0.349]
	SD	0.092	25.3	[0.026; 0.151]
**CL12(L/h)**	Mean	9.960 (fixed)		
	SD	0		
**V2(L/kgLBMc)**	Mean	0.686		[0.482; 1.087]
	SD	0.335	30.4	[0.130; 0.604]
**Eec(-)**	Mean	0.212		[0.126; 0.281]
	SD	0.069	63.7	[0.039; 0.091]

The nonparametric 95% CI is obtained by bootstrapping with 1000 repetitions.

### Model evaluation

**Fig 3:**
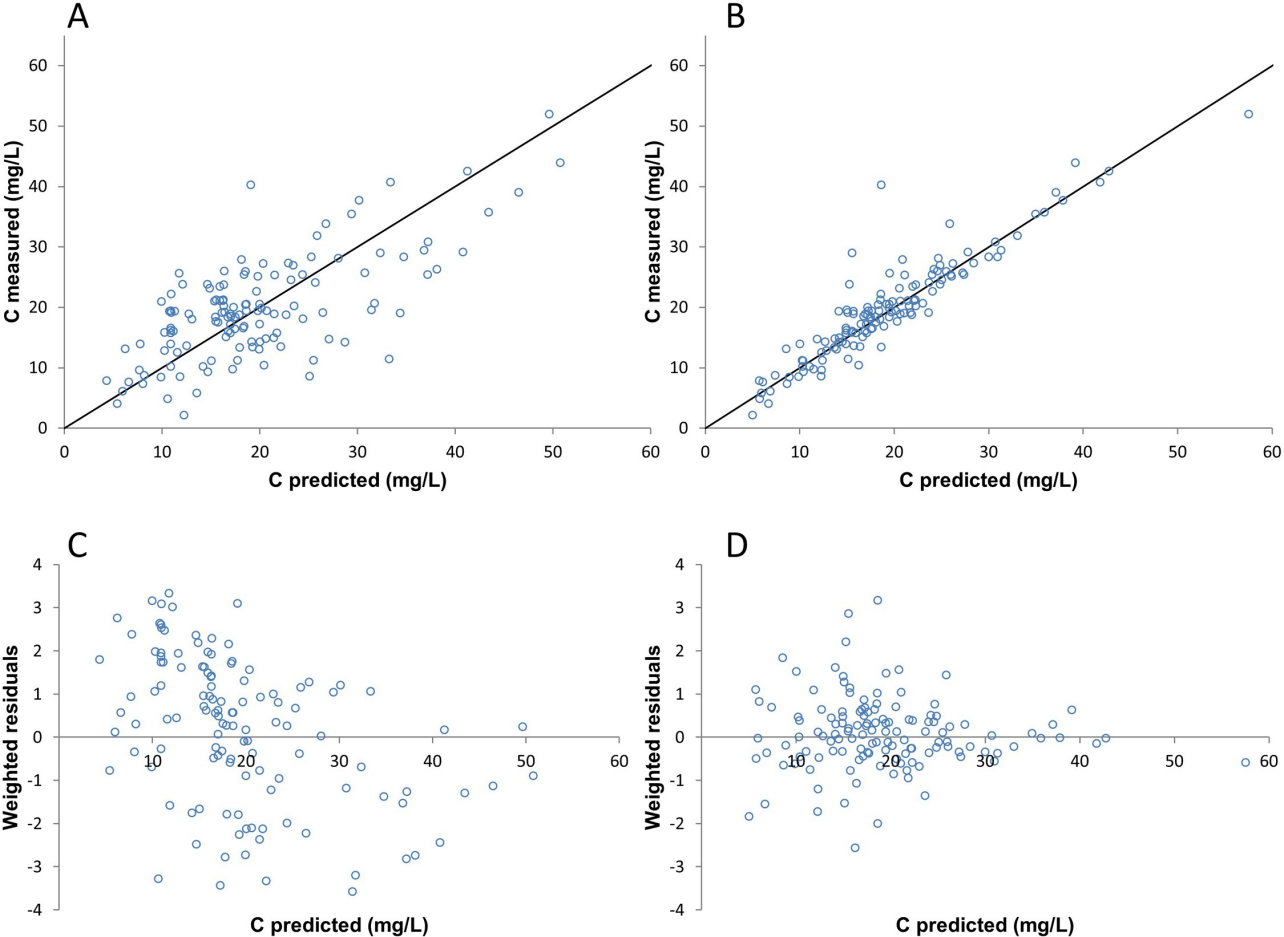
Diagnostic goodness of fit and weighted residuals plots of the evaluation cohort (LF-HD patients). (A) Population predicted vancomycin serum levels based on the final model versus the actually measured vancomycin serum levels. (B) Individual predicted vancomycin serum levels based on the final model versus the actually measured vancomycin serum levels. (C) Weighted residuals of the population predicted vancomycin serum levels versus the predicted vancomycin serum levels. (D) Weighted residuals of the individual predicted vancomycin serum levels versus the predicted vancomycin serum levels.

The individual predicted vancomycin concentrations in Fig 3B are closer distributed along the line of identity compared to the population predicted vancomycin concentrations in Fig 3A (LF-HD patients). MDPE is -0.7% (95% CI: -3.4%-1.7%) and the MDAPE as a measure of precision is 7.9% (95% CI: 6.0%-9.8%), for the individual predicted vancomycin concentrations. The weighted residuals for the population predicted vancomycin concentrations in Fig 3C look to be skewed, more data points are under the y = 0 line above a predicted concentration of 30 mg/L. The weighted residuals for the individual predicted vancomycin concentrations in Fig 3D appear to be distributed evenly along the line of identity.

### Dosing regimen

In Tables 3 and 4 the calculated ‘a priori’ dosing regimen to achieve AUC_24h_ ≥400mg*h/L are shown for any patient on LF-HD or HDF starting with vancomycin. In Table 3 the dosing regimen has a dosing interval of 48 hours and in Table 4 the dosing regimen has a dosing interval of 72 hours. In both HDF and LF-HD, the optimal vancomycin loading dose for a patient weighing 70 kg is 1700 mg if administered during the last 60 minutes of the hemodialysis session. The optimal maintenance dose is 700 mg if administered during the last 30 or 60 minutes of the hemodialysis session for a patient weighing 70 kg with a dosing interval of 48 hours.

**Table 3:** Dosing regimen for administering vancomycin during HDF and LF-HD using a dosing interval of 48 h.

	infused during last 60 min			Infused during last 60 min			Infused during last 30 min		
	Extracorporeal bloodflow (mL/min)			Extracorporeal bloodflow (mL/min)			Extracorporeal bloodflow (mL/min)		
	200	250	300	200	250	300	200	250	300
Weight (kg)	Loading dose (mg)			Maintenance doses (mg)					
**50**	1300	1300	1400	700	800	800	700	700	700
**60**	1500	1500	1500	700	700	800	700	700	700
**70**	1600	1700	1700	700	700	800	700	700	700
**80**	1700	1700	1700	700	700	800	700	700	700
**90**	1800	1800	1800	700	700	800	700	700	700
**100**	1800	1900	1900	700	700	800	700	700	700
**110**	1900	1900	1900	700	700	800	700	700	700
**120**	2000	2000	2000	700	700	800	700	700	700

The dosing regimen is based on infusion during the final 60 min versus the final 30 min of HDF and LF-HD for different weight classes and different extracorporeal bloodflows to achieve AUC_24h_≥400 mg*h/L. Vancomycin dosages were rounded up to a multiple of 100 mg. This dosing regimen for dosing vancomycin is applicable to HDF and LF-HD patients.

**Table 4:** Dosing regimen for administering vancomycin during HDF and LF-HD using a dosing interval of 72 h.

	infused during last 60 min			Infused during last 60 min		
	Extracorporeal bloodflow (mL/min)			Extracorporeal bloodflow (mL/min)		
	200	250	300	200	250	300
Weight (kg)	Loading dose (mg)			Maintenance doses (mg)		
**50**	1700	1700	1700	1000	1000	1100
**60**	1800	1800	1800	1000	1000	1000
**70**	1900	2000	2000	1000	1000	1000
**80**	2000	2000	2000	1000	1000	1000
**90**	2100	2100	2100	1000	1000	1000
**100**	2100	2100	2200	1000	1000	1000
**110**	2200	2200	2200	1000	1000	1000
**120**	2200	2300	2300	1000	1000	1000

The dosing regimen is based on infusion during the final 60 min of HDF and LF-HD for different weight classes and different extracorporeal blood flows to achieve AUC_24h_≥400 mg*h/L. Vancomycin dosages were rounded up to a multiple of 100 mg. This dosing regimen for dosing vancomycin is applicable to HDF and LF-HD patients.

## Discussion

To our knowledge this is the first study that has developed a PPK model for administration of intravenous vancomycin in HDF patients, evaluates this regimen in LF-HD patients and provides an ‘a priori’ dosing regimen for administration during HDF and LF-HD. The relatively small study populations precludes to draw firm conclusions about a difference between the pharmacokinetics in HDF and LF-HD. However we showed that our developed HDF PPK model also adequately predicts the serum concentrations in LF-HD patients in a clinical setting.

The goodness-of-fit plot of the individual predicted vancomycin concentrations in Fig 2B shows that the final model predicts the vancomycin serum levels in HDF patients adequately. The bootstrap analysis showed that the final model is robust. The goodness-of-fit plot in Fig 3B shows an even distribution of data points closely along the line of identity and shows that the developed PPK model also predicts the vancomycin serum levels in LF-HD patients adequately. The novel HDF PPK model is therefore also useful for LF-HD patients. Jager et al. stated that it is likely that the vancomycin clearance is increased in HDF patients compared to HD patients, however, our study could not find a difference in predicting vancomycin concentrations for HDF or LF-HD patients based upon our PPK model[3]. It is still possible that there is a higher clearance in HDF patients, but that difference could not be found in this study, e.g. due to the retrospective nature of this study and the small population.

The V1 found in our study was 19.5 L and V2 was 48.0 L, Ghouti-Terki et al. found comparable values of 15.4 L and 62.3 L respectively for V1 and V2[9]. The CLm is higher than found in previous studies, 0.473 L/h compared to 0.29 L/h respectively[9]. This is probably because of the fixed fr, because fr and CLm are correlated according to Eq 2. On the other hand, the CLm (0.473 L/h (7.9 ml/min)) we found in this study is close to the range of 5–6 ml/min reported by Launay-Vacher et al[7].

The dataset contained not enough samples shortly after vancomycin administration, therefore the CL12 could not be estimated accurately and was fixed at a literature value. The fr could not be estimated properly because the CLcr is low (on average 4.4 ml/min) and thus the renal clearance (fr * CLcr) does not contribute much to the total elimination.

Ghouti-Terki et al. found a vancomycin dialysis clearance of 134 L/day in LF-HD patients[9], the vancomycin dialysis clearance of 74 L/day we found in our study for HDF patients is considerably lower. However if the Eec for the study of Ghouti-terki et al. is calculated to correct for a higher extracorporeal bloodflow in that study according to Eq 2, the Eec is 26.8%, which is close to the Eec of 21.2% found in our study. The η-shrinkage for the Eec is high (63.9%), indicating that the individual estimates of Eec are biased towards the population mean[16]. The higher Eec found by Ghouti-Terki et al. can possibly be explained by more efficient high-flux dialysis membranes in that study, compared to the FX80 high-flux filters (development cohort, HDF patients) used in our study. The relatively high η-shrinkage for Eec may be due to the simultaneous efflux (dialysis clearance) and influx (vancomycin administration) and lack of information about the ratio between the efflux and influx.

Based on the new developed PPK model an ‘a priori’ dosing regimen was designed and is shown in Tables 3 and 4. The maintenance dose is almost independent of body weight (Tables 3 and 4), due to the fact that the clearance, and consequently AUC, is determined by Qec and Eec, which are independent of body weight in our simulations. There is a slight trend that the maintenance dose decreases with increasing body weight. This apparent anomaly is due to our worst-case calculation of AUC. Increased body weight is associated with a larger volume of distribution and consequently results in a longer half-life, and a higher AUC during the last 24 hour of the dosing interval, and thus a lower maintenance dose. The total AUC over the entire dosing interval of 48 or 72 hours is independent of body weight.

In Tables 3 and 4 it can be seen that the loading dose increases less than proportional with body weight. This may be explained by considering that the loading dose is the sum of (1) the amount of vancomycin needed to fill the volume of distribution, which is close to proportional to body weight, and (2) the amount of vancomycin dialysed, which is (almost) independent of body weight, since it is determined by the vancomycin concentration, Qec and Eec. The increase in dose of vancomycin during the final 60 versus the final 30 minutes was rather small, and the extracorporeal bloodflows also had little effect on the dosing during dialysis.

Ghouti-Terki et al. found that the dose of vancomycin for a typical patient administered during the last 60 minutes of dialysis is 1400 mg[9], this dose is substantially higher than our dosing regimen. However, it is not clear if the reported dose of 1400 mg is a loading or a maintenance dose administered during hemodialysis[9]. All our patients were dialyzed over a period of 4 hours, in the study of Ghouti-Terki et al. this varied between 4 and 5 hours[9]. To simulate vancomycin exposures Ghouti-Terki et al. assumed a dialyzing period of 4.5 and a target AUC_24h_ of 400 mg*h/L. Because of the increased dialysis clearance and increased dialyzing period (4.5 h compared to 4 h in our study), more vancomycin was cleared by the dialyzer in the study of Ghouti-Terki et al. This partly explains the higher dose of vancomycin compared to our study.

Our study, however, has some limitations: 1) a small number of patients, 2) MIC levels were not available and 3) the retrospective nature of our study. Because of the small number of patients we were not able to do a full covariate analysis. In clinical practice vancomycin is used up till a MIC level of 1 mg/L and therefore we targeted at an AUC_24h_≥400 mg*h/L.

### Conclusion and recommendations

A PPK model for vancomycin during HDF was developed, this PPK model also reliably predicts serum levels of vancomycin in LF-HD patients. Based on this new PPK model an ‘a priori’ dosing regimen was developed for the use in HDF and LF-HD patients based on weight and extracorporeal bloodflows.

Further perspectives may include covariate analysis of factors influencing the pharmacokinetics of vancomycin and prospectively validating the newly developed dosing regimen. Covariates that can be considered are different dialysis filter types, however, this implies the study of a larger population.

## Supporting information

S1 Data Demographics. (XLSX)

S2 Data Individual and population predicted and measured concentrations. (XLSX)

S3 Data MDPE and MDAPE calculation by bootstrapping with 10 000 repetitions. (XLS)
